# Isometric yoga improves the fatigue and pain of patients with chronic fatigue syndrome who are resistant to conventional therapy: a randomized, controlled trial

**DOI:** 10.1186/s13030-014-0027-8

**Published:** 2014-12-11

**Authors:** Takakazu Oka, Tokusei Tanahashi, Takeharu Chijiwa, Battuvshin Lkhagvasuren, Nobuyuki Sudo, Kae Oka

**Affiliations:** Department of Psychosomatic Medicine, Graduate School of Medical Sciences, Kyushu University, Fukuoka, 812-8582 Japan; Department of Pediatrics and Child Health, School of Medicine, Kurume University, Asahi-machi 67, Kurume, 830-0011 Japan

**Keywords:** Chronic fatigue syndrome, Isometric yoga, Fatigue, Treatment, Fibromyalgia

## Abstract

**Background:**

Patients with chronic fatigue syndrome (CFS) often complain of persistent fatigue even after conventional therapies such as pharmacotherapy, cognitive behavioral therapy, or graded exercise therapy. The aim of this study was to investigate in a randomized, controlled trial the feasibility and efficacy of isometric yoga in patients with CFS who are resistant to conventional treatments.

**Methods:**

This trial enrolled 30 patients with CFS who did not have satisfactory improvement after receiving conventional therapy for at least six months. They were randomly divided into two groups and were treated with either conventional pharmacotherapy (control group, n = 15) or conventional therapy together with isometric yoga practice that consisted of biweekly, 20-minute sessions with a yoga instructor and daily in-home sessions (yoga group, n = 15) for approximately two months. The short-term effect of isometric yoga on fatigue was assessed by administration of the Profile of Mood Status (POMS) questionnaire immediately before and after the final 20-minute session with the instructor. The long-term effect of isometric yoga on fatigue was assessed by administration of the Chalder’s Fatigue Scale (FS) questionnaire to both groups before and after the intervention. Adverse events and changes in subjective symptoms were recorded for subjects in the yoga group.

**Results:**

All subjects completed the intervention. The mean POMS fatigue score decreased significantly (from 21.9 ± 7.7 to 13.8 ± 6.7, *P* < 0.001) after a yoga session. The Chalder’s FS score decreased significantly (from 25.9 ± 6.1 to 19.2 ± 7.5, *P* = 0.002) in the yoga group, but not in the control group. In addition to the improvement of fatigue, two patients with CFS and fibromyalgia syndrome in the yoga group also reported pain relief. Furthermore, many subjects reported that their bodies became warmer and lighter after practicing isometric yoga. Although there were no serious adverse events in the yoga group, two patients complained of tiredness and one of dizziness after the first yoga session with the instructor.

**Conclusions:**

Isometric yoga as an add-on therapy is both feasible and successful at relieving the fatigue and pain of a subset of therapy-resistant patients with CFS.

**Trial registration:**

University Hospital Medical Information Network (UMIN CTR) UMIN000009646.

## Background

Chronic fatigue syndrome (CFS) is a debilitating disease characterized by persistent fatigue that is not relieved by rest and by other nonspecific symptoms, all of which last for a minimum of six months [1]. The pathophysiological mechanisms underlying CFS are not yet fully understood. Currently, patients with CFS are treated with antidepressants, cognitive behavioral therapy (CBT), and/or graded exercise therapy (GET) [2–5]. However, there are patients who do not fully recover even with these treatments.

Yoga is one of the most commonly accepted mind/body therapies of complementary and alternative medicine and is recommended as an alternative therapy for improving unexplained chronic fatigue [6]. In fact, several studies have demonstrated that yoga is effective in improving the fatigue of patients with cancer [7,8] as well as of healthy subjects [9]. We hypothesized that yoga is also effective in improving the fatigue of patients with CFS. However, as the yoga programs practiced in previously published studies were not uniform, it was difficult to identify which program or which component of yoga is useful for alleviating fatigue. Furthermore, patients with CFS complain of severe fatigue, especially after exertion. Therefore, before starting this study, we discussed the yoga program with yoga instructors to determine which type of practice had the least probability of exacerbating a patient’s fatigue. We selected isometric yoga, as described in the methods section.

The aims of this study were to assess the feasibility of isometric yoga among patients with CFS and to assess the effect of isometric yoga on fatigue and related psychological and physical symptoms of patients with CFS who did not respond to conventional therapies. To our knowledge, this is the first study to investigate the effect of isometric yoga on the fatigue of patients with CFS.

## Methods

This study was approved by the Institutional Review Board of Kyushu University. Written informed consent was obtained from all participants before they were enrolled.

### Subjects

This study enrolled outpatients with CFS who visited the Department of Psychosomatic Medicine of Kyushu University Hospital. Inclusion criteria were the following: (1) the subject’s fatigue did not improve sufficiently with ordinary treatment given in our Department (as an example see [10]), including pharmacotherapy (for example, antidepressants, Japanese traditional herbal medicine [11,12], and/or coenzyme Q10), psychotherapy, and/or GET; in some cases, a four-week inpatient treatment program was also included [10]) for at least six months; (2) the subject was between 20 and 70 years old; (3) the subject’s level of fatigue was serious enough to cause an absence from school or the workplace at least several days a month but not serious enough to require assistance with the activities of daily living; (4) the subject was able to fill out the questionnaire without assistance; (5) the subject could sit for at least 30 minutes; and (6) the subject could visit Kyushu University Hospital regularly every two or three weeks. Subjects were excluded if (1) their fatigue was due to a physical disease such as liver, kidney, heart, respiratory, endocrine, autoimmune, or malignant disease, severe anemia, electrolyte abnormalities, obesity, or pregnancy; and (2) they had previously practiced yoga. The diagnosis of CFS was made for patients meeting the diagnostic criteria of the 1994 international research case definition of CFS [1]. Patients with idiopathic chronic fatigue were not included in this study.

### Methods

**Figure 1:**
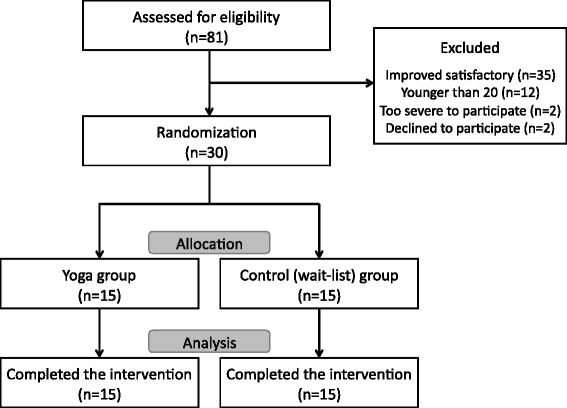
**Flow chart outlining participation in this study.**

Following enrollment, eligible participants were randomized using a computer-generated randomization list to receive either an isometric yoga practice together with conventional pharmacotherapy group (yoga group, n = 15) or to a conventional pharmacotherapy alone group (wait-list control group, n = 15) for approximately two months. As the patients visited the hospital every two or three weeks, the intervention period lasted 9.2 ± 2.5 (mean ± standard deviation (SD)) weeks after the start of the intervention (Figure 1).

### Development of the yoga program

**Figure 2:**
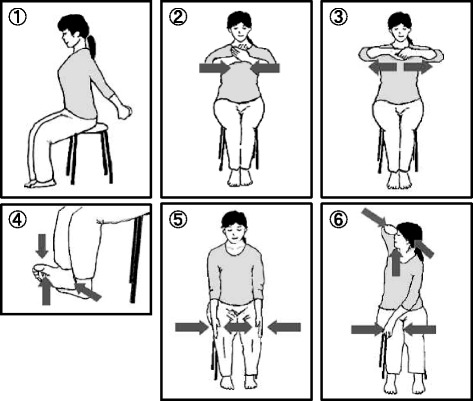
**Illustration of the six poses of the isometric yoga program used in this study.** The 20-minute isometric yoga program consisted of three parts. **1)** The patients practiced being aware of their spontaneous breathing for one minute. **2)** The patients practiced six isometric poses **4–6** times: (i) stretching both arms behind the back; (ii) pushing the palms against each other; (iii) pulling the palms away from each other; (iv) pushing the feet against each other; (v) pushing both knees inward with hands on the outside; and (vi) twisting. **3)** The patients practiced abdominal breathing for one minute. The postures were practiced slowly in association with exhalation or inhalation with 50% of the maximal physical strength. After the postures were repeated **4–6** times, the patients decreased their physical exertion and returned slowly to the basic position while exhaling.

Before starting this trial, we consulted yoga instructors to identify a program that would satisfy the following requirements. Firstly, because patients with CFS have severe fatigue, it should not exacerbate their symptoms or cause post-exertion malaise. Secondly, because the patients are deconditioned, it should also act as an exercise therapy. Thirdly, because the patients’ concentration and short-term memory are impaired, it should be simple and easy to do. Fourthly, because the patients would be treated at the hospital, not at a yoga studio, it must be able to be practiced in an outpatient setting, where space is limited. To satisfy these requirements, we determined that the trial would include isometric yoga, or an isometric yogic breathing exercise, as a treatment for patients with CFS. Isometric yoga, which was developed by Dr. Keishin Kimura, differs from traditional yoga postures in several ways. The predominant difference is that the poses consist mainly of isometric muscle contractions. Since the patients can change resistance depending on their fatigue level, we thought that isometric yoga would help prevent worsened fatigue. These poses do not include isotonic muscular contractions or strong stretching and require less physical flexibility. Therefore, we hypothesized that practicing this form of yoga would be easy on the patients, preventing over-stretching, which is detrimental and may increase pain. However, as are traditional yoga poses, these poses are conducted slowly in accordance with breathing and with awareness of inner sensations. We intentionally avoided standing postures, because a considerable number of CFS patients suffer from orthostatic intolerance, including postural orthostatic tachycardia syndrome [13]. This 20-minute yoga program can be practiced in a sitting position and consists of three parts. First, patients are asked to be aware of their spontaneous breathing for one minute. Next, they practice six poses. These poses are very slow movements that are coordinated with the timing of breathing, with or without sounds, and isometric exercise at 50% of the patient’s maximal physical strength. Lastly, the patients practice abdominal breathing for one minute (Figure 2).

### Yoga intervention

Patients in the yoga group practiced isometric yoga in a quiet room for 20 minutes on a one-to-one basis with an instructor who has over 30 years of experience. The sessions occurred between 2 pm and 4 pm on the day they visited the hospital. In this program, the yoga instructor was not allowed to use background music, which is often used in the yoga studio to facilitate the participants’ relaxation, because many patients with CFS are sensitive to sounds. Before and after practicing isometric yoga, the doctors in charge checked the patient’s condition and recorded any adverse events or any changes caused by practicing isometric yoga. In addition to receiving a private lesson, the participants were asked to practice this program on non-class days if they could, with the aid of a digital videodisc and a booklet. Most patients visited their doctor every two to three weeks during the intervention period. Therefore, all patients practiced isometric yoga at least four times (mean ± SD, 5.6 ± 1.7 times) with the instructor during the intervention period. Basically, all patients practiced the same 20-min program, both with an instructor and at home. However, the program was modified on a patient-to-patient basis, in most cases skipping a certain pose or decreasing the number of repetitions of poses, depending on the severity of their fatigue and the pain associated with the pose.

### Outcome assessment

To assess the acute effects of isometric yoga, the fatigue (F) and vigor (V) scores of the Profile of Mood States (POMS) questionnaire [14] were assessed immediately before and after the final 20-minute session of isometric yoga with the instructor. To assess the chronic effects of isometric yoga, fatigue was assessed with Chalder’s fatigue scale (FS) [15] before and after the intervention period in the yoga and control groups. Chalder’s FS is a well-validated, self-reported scale that measures the physical and mental symptoms of fatigue. In the yoga group, the assessments were conducted just before practicing yoga. Patients in this group also completed the Medical Outcomes Study Short Form 8, standard version (SF-8™) before and after the intervention period to assess their health-related quality of life (QOL) [16]. Questionnaires were collected by a nurse.

### Adverse events and adherence

Adverse events were monitored in two ways. First, at each visit to the hospital, the doctors in charge determined if a subject experienced any uncomfortable symptoms after practicing yoga with the instructor. Second, patients in the yoga group were asked to keep a “yoga diary,” in which they could record the amount of time they practiced and how they felt after practicing yoga. On the day of the visit, before the patient practiced yoga with the instructor, the doctors checked the diary and determined if the patient had had any symptoms of discomfort. After the intervention period, the diary was collected and checked to determine how often the subjects had practiced yoga at home.

### Statistical analyses

The data are presented as the mean ± SD. The differences in the outcome measures were tested by two-way, repeated measures, analysis of variance (ANOVA) of the mean scores. Two comparisons were made: one compared the scores of the yoga group to those of the control group; the other compared the scores measured before the intervention to those measured after. The differences in the patients’ POMS, Chalder’s FS, and SF-8 scores measured before and after the intervention were tested by use of a paired-sample *t* test. Between-group differences in age and in the POMS, Chalder’s FS, and SF-8 scores measured before and after the intervention were tested by use of an independent-sample *t* test. Two-tailed tests were used. Fisher’s exact probability test was also used when appropriate. Data were analyzed by using SPSS for Windows, V.17.

## Results

### Participants

The study comprised 30 subjects, with 15 in the yoga group (age range: 24–60 years; mean age (mean ± SD): 38.0 ± 11.1 years; 3 men) and 15 in the control group (age range: 20–59 years; mean age: 39.1 ± 14.2 years; 3 men). All patients completed the study. There were no significant differences in age, sex, or Chalder’s FS total and subscale scores measured before the intervention between the yoga group and the control group (Table 1). The mean Chalder’s FS score at the first hospital visit of both groups was 30.8 ± 4.5.

**Table 1:** **Demographic characteristics of the participants**

	**Yoga group**	**Control group**
Number(m:f)^#^	15(3:12)	15(3:12)
Age (years)*	38.0 ± 11.1	39.1 ± 14.2
Chalder’s fatigue scale, Physical fatigue*	16.4 ± 3.5	16.5 ± 3.4
Chalder’s fatigue scale, Mental fatigue*	9.5 ± 3.5	9.7 ± 3.2
Chalder’s fatigue scale, Total score*	25.9 ± 6.1	26.1 ± 6.2

The data shown are the mean ± standard deviation (SD). #Not significant by the Fisher exact probability test. *Not significant by an independent-sample *t* test.

### Short-term effects of isometric yoga on fatigue and vigor

**Figure 3:**
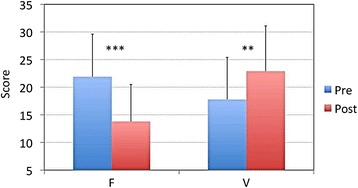
**Acute effects of isometric yoga on fatigue and vigor.** A comparison of the fatigue (F) and vigor (V) scores of the Profile of Mood States (POMS) questionnaire for participants in the yoga group before (pre, blue) and immediately after (post, red) the final 20-minute session of isometric yoga with the instructor. ***P < 0.001, **P < 0.01 (paired *t* test).

We assessed the short-term effects of isometric yoga on fatigue by comparing the POMS F and V scores before and after the patients completed the final 20-minute session of isometric yoga with the instructor. We used the POMS because the Chalder’s FS is not appropriate for evaluating short-term changes in fatigue. Practicing isometric yoga significantly decreased the mean F score (from 21.9 ± 7.7 to 13.8 ± 6.7, *P* < 0.001) and increased the mean V score (from 17.8 ± 7.6 to 22.9 ± 8.2, *P* = 0.002) (Figure 3).

### Long-term effects of isometric yoga on fatigue

To assess the long-term effects of regular practice of isometric yoga on fatigue, we compared the Chalder’s FS total score and the subscale scores for physical and mental symptoms of the control group to those of the yoga group before and approximately two months after the intervention. At baseline, the three scores did not differ significantly between the two groups.

**Figure 4:**
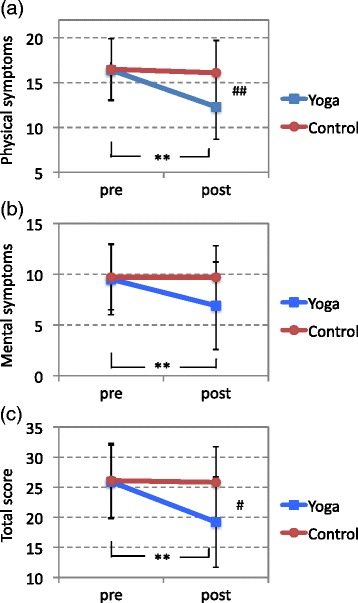
**Chronic effects of isometric yoga on fatigue.** Chalder’s Fatigue Scale (FS) subscale scores for physical symptoms **(a)**, mental symptoms **(b)**, and FS total scores **(c)** of the yoga and control groups. **P < 0.01 for differences between the pre- and post-intervention scores (paired-sample *t* test). # *P* < 0.05, ## *P* < 0.01 for differences in the scores between the yoga and control groups (independent-sample *t* test).

We observed a significant main effect of time in the repeated measure ANOVA for the total score and for the two subscores (total score; f(1) = 12.4, *P* = 0.001: physical symptoms subscore; f(1) = 11.0, *P* = 0.002: and mental symptoms subscore; f(1) = 8.6, *P* = 0.007). We also found a significant interaction between the intervention and time (total score; f(1) = 10.2, *P* = 0.003: physical symptoms subscore; f(1) = 7.9, *P* = 0.009: and mental symptoms subscore; f(1) = 8.6, *P* = 0.007) (Figure 4 and Table 2). This finding indicates that patients who practiced yoga experienced a greater improvement in fatigue than did those who did not. The main effect for group was not significant for any score (total score; f(1) = 2.5, *P* = 0.124: physical symptoms subscore; f(1) = 2.9, *P* = 0.099: and mental symptoms subscore: f(1) = 1.4, *P* = 0.252).

**Table 2:** **Changes in Chalder**’**s Fatigue Scale** (**FS**) **subscale and total scores from baseline to post**-**intervention of patients in the yoga group compared with the score changes of those in the control group**

**Chalder** **’** **s fatigue scale**	**Yoga group mean** **(SD)**	**Control group mean** **(SD)**	**Significance of change** **(P values)** **Within** **-** **group**		**Time x group interaction**
			**Yoga group**	**Control group**	
Physical symptoms					
Pre-intervention	16.4 (3.5)	16.5 (3.4)	0.004	0.779	0.009
Post-intervention	12.3 (3.8)	16.1 (3.6)			
Mental symptoms					
Pre-intervention	9.5 (3.5)	9.7 (3.2)			
Post-intervention	6.9 (4.4)	9.7 (3.1)	0.004	0.395	0.007
Total score					
Pre-intervention	25.9 (6.1)	26.1 (6.2)			
Post-intervention	19.2 (7.5)	25.8 (5.9)	0.002	0.550	0.003

Tested with repeated measures analysis of variance (ANOVA), followed by post-hoc, within-group, paired-sample *t* tests.

At the time of the post-intervention evaluation, both the physical symptoms subscale score and the total score of the yoga group were significantly lower than those of the control group (*P* = 0.005, *P* = 0.022, respectively by the independent-sample *t* test). In the control group, neither the total score nor the two subscores differed significantly between the pre- and post-intervention evaluations. In contrast, in the yoga group the mean physical and mental symptoms subscores and the mean FS total score decreased significantly after the intervention period (*P* = 0.004, *P* = 0.004, *P* = 0.002, respectively, by the paired-sample *t* test; Figure 4).

### Effect of regular practice of isometric yoga on health-related QOL

To assess if the regular practice of yoga affects health-related QOL, we compared the SF-8^TM^ scores of participants in the yoga group before and after the intervention. At baseline, all subscale scores were less than 50, suggesting that the QOL of patients with CFS is lower than that of the average Japanese population. Among the 10 subscale scores, the mean scores for three increased significantly after regular practice of isometric yoga: bodily pain (BP, from 41.3 ± 6.7 to 48.1 ± 7.9, *P* = 0.0001), general health (GH, from 39.3 ± 5.3 to 43.6 ± 6.0, *P* = 0.002), and physical component summary (PCS, from 35.8 ± 7.2 to 40.6 ± 4.7, *P* = 0.024) (Table 3). These data suggest that yoga relieved the patients’ pain and improved their general health.

**Table 3:** **Changes in Short**-**Form 8** (**SF**-**8**) **scores obtained before** (**pre**-) **and after** (**post**-) **the intervention for participants in the yoga group**

	**Pre**	**Post**	**P value**
Physical functioning (PF)	39.6 ± 9.1	42.5 ± 7.1	n.s.
Role physical (RP)	34.4 ± 8.4	38.4 ± 6.4	n.s.
Bodily pain (BP)	41.3 ± 6.7	48.1 ± 7.9	P = 0.0001
General health perception (GH)	39.3 ± 5.3	43.6 ± 6.0	P = 0.0021
Vitality (VT)	43.7 ± 4.9	43.5 ± 6.1	n.s.
Social functioning (SF)	37.6 ± 7.8	37.6 ± 7.8	n.s.
Role emotional (RE)	39.2 ± 12.6	44.4 ± 9.3	n.s.
Mental health (MH)	45.8 ± 9.5	46.8 ± 9.5	n.s.
Physical component summary (PCS)	35.8 ± 7.2	40.6 ± 4.7	P = 0.024
Mental component summary (MCS)	44.1 ± 8.5	44.5 ± 7.9	n.s.

The data shown are the mean ± standard deviation (SD). The *P* values assess the differences in scores between the pre- and post-intervention periods (paired-sample *t* test).

### Safety and adverse events

At the hospital, one female subject complained of dizziness after her first yoga session. However, she did not require any specific treatment and she did not experience any negative symptoms in the subsequent sessions. After the first yoga session, two patients reported that they felt tired because they had to concentrate and follow the instructions. In subsequent sessions, neither reported that they experienced this symptom. None of the other patients reported any adverse symptoms. During the home practice, two patients reported that they felt light-headed when they practiced yoga with their eyes closed or on bad days, but not with their eyes open or on good days. Finally, none of the patients reported disabling post-exertion malaise after they practiced yoga.

### Other patient reported outcomes

The short-term effects commonly reported by the patients after practicing isometric yoga included a feeling of warmth (n = 11) and lightness (n = 8). One subject mentioned that these benefits lasted for one hour. Seven patients reported that they felt more calm, relaxed, and worry-free. Five patients reported pain relief during their yoga practice. Two patients who have fibromyalgia syndrome as well as CFS skipped the arm-stretching pose because it was difficult and painful for them. They reported that, in the beginning, practicing yoga caused a transient increase in pain because they had to focus on the inner sensations of their bodies. However, as their practice proceeded, they were able to become detached from the pain, and they noticed that the severity of the pain decreased during a yoga session. Regarding the long-term effects, seven patients reported that they now noticed how tense they were in their daily lives and how helpful it was to release muscular tension during yoga. Two patients reported “After regular practice of yoga, I started to wake up more easily in the morning, which had been hard, because tiredness in the morning decreased.”

### Adherence

Adherence was very good overall. All patients practiced yoga with an instructor when they visited the hospital. Fourteen of the 15 patients kept yoga diaries. Based on the records in their diaries, these 14 patients practiced isometric yoga at home for a mean of 5.8 ± 1.8 days/week and 5.7 ± 1.8 days/week during the first and last weeks of the intervention period, respectively.

### Satisfaction

Fourteen of the 15 patients cited high satisfaction and described isometric yoga as being useful and helpful. One subject reported that she did not want to continue her yoga practice after the intervention period because she was afraid to close her eyes and to see inside.

## Discussion

The present study demonstrated that isometric yoga together with conventional therapy was more effective in relieving fatigue than was conventional therapy alone in patients with CFS who did not respond adequately to conventional therapy. To our knowledge, this is the first clinical trial that assessed the effects of yoga on the fatigue of CFS patients.

The first aim of this study was to assess the feasibility of isometric yoga as a treatment for CFS. Some patients with CFS experience adverse events such as worsening of fatigue and physical function even when treated with conventional therapies such as CBT and GET [17]. Although two patients in this study complained of tiredness at the first yoga session, they did not have this complaint after they became accustomed to the procedures. Participants had neither serious adverse events nor post-exertion malaise lasting for more than 24 hours. Furthermore, this study exhibited an excellent level of adherence and participant satisfaction. Taken together, our results suggest that an isometric yoga program is both feasible and acceptable for patients with CFS.

Our results also indicate that isometric yoga can significantly improve fatigue, enhance vigor, reduce pain, and improve QOL, and thus may offer a promising new treatment modality for patients with therapy-resistant CFS. The isometric yoga intervention reduced Chalder’s FS scores, especially the physical symptoms subscore. It also improved the BP, GH, and PCS subscores of the SF-8, although it did not improve the mental component summary subscore. Therefore, isometric yoga may improve the physical components of CFS, including physical fatigue or pain, more effectively than the psychological ones. Interestingly, isometric yoga improved pain as well as fatigue. In the yoga group, two patients who were diagnosed with both CFS and fibromyalgia syndrome also reported pain relief. Therefore, isometric yoga might be an effective treatment for both conditions.

Some patients reported these benefits even just after their first session with an instructor. In contrast, others reported adverse events such as dizziness in the beginning. For some patients, it was an effort to memorize the procedures. However, as they practiced, they eventually felt the beneficial effects described above. In most cases, the patients began to feel beneficial effects within one month of practicing isometric yoga, as determined from their yoga diary and interviews.

Previous studies have demonstrated that yoga improves the fatigue of patients with cancer [7]. Although the precise mechanisms of cancer-related fatigue are not yet fully understood, several common mechanisms are suggested to exist between cancer-related fatigue and the fatigue associated with CFS. These include dysfunction of the autonomic nervous system and the hypothalamic-pituitary-adrenal axis, disruption of circadian rhythms, and misregulation of cytokine expression [18–23]. Yoga has been reported to reduce serum levels of cortisol [24] and proinflammatory cytokines such as interleukin-6 [25,26]. It also increases heart rate variability and shifts the autonomic nervous system from a state predominated by sympathetic activity to one predominated by parasympathetic activity [27,28]. All of these changes may contribute to the beneficial effects of isometric yoga, one of which is reduced fatigue. As many patients reported that their bodies became warmer during a yoga session, isometric yoga may improve systemic circulation, and this physiological change might also reduce the pain and fatigue of CFS. However, the mechanisms behind the beneficial effects of this isometric yoga program are not fully understood yet. Therefore, these will be the focus of a future study. We have already investigated the changes in autonomic functions and in the blood levels of several biomarkers, the results of which will be published soon.

### Limitations

This study had several limitations. Firstly, the effects of yoga were evaluated for patients (1) whose fatigue level was not serious enough to require assistance with the activities of daily living, and (2) whose fatigue did not recover fully with ordinary treatment for more than six months. Secondly, we customized the yoga program for the patients in this study. Therefore, further studies are necessary to determine if we can generalize these findings to all patients with CFS or to any kind of yoga program. Thirdly, as this study evaluated the feasibility of a yoga program, we assessed the effects of isometric yoga for a relatively small number of patients and for a short intervention period. Two months might not be sufficient to fully evaluate the feasibility and the effects of yoga. Future studies should evaluate the long-term effects of isometric yoga. Finally, one subject did not want to continue practicing isometric yoga after the intervention period. This patient experienced psychological trauma at a young age and had a difficult life after that. Therefore, future studies should evaluate and treat comorbid psychiatric diseases, especially posttraumatic stress disorder, before considering treatment with yoga.

## Conclusions

This study demonstrated that a combination therapy consisting of isometric yoga and pharmacotherapy is feasible and that it can relieve the fatigue and pain of patients with CFS who are resistant to conventional therapy. Further studies are needed to determine the mechanisms by which isometric yoga improves fatigue.

## Abbreviations

ANOVA: Analysis of variance
BP: Bodily pain
CFS: Chronic fatigue syndrome
CBT: Cognitive behavioral therapy
F: Fatigue
GET: Graded exercise therapy
GH: General health perception
MCS: Mental component summary
MH: Mental health
SD: Standard deviation
SF: Social functioning
SF-8: Short form-8
PF: Physical functioning
PCS: Physical component summary
POMS: Profile of mood states
QOL: Quality of life
RE: Role emotional
RP: Role physical
V: Vigor
VT: Vitality
